# Identification of Potential Blind-Side Hypermelanosis-Related lncRNA–miRNA–mRNA Regulatory Network in a Flatfish Species, Chinese Tongue Sole (*Cynoglossus semilaevis*)

**DOI:** 10.3389/fgene.2021.817117

**Published:** 2022-02-03

**Authors:** Yangzhen Li, Yuanri Hu, Peng Cheng, Songlin Chen

**Affiliations:** ^1^ Shandong Key Laboratory of Marine Fisheries Biotechnology and Genetic Breeding, Yellow Sea Fisheries Research Institute, Chinese Academy of Fishery Sciences, Qingdao, China; ^2^ Laboratory for Marine Fisheries Science and Food Production Processes, Pilot National Laboratory for Marine Science and Technology (Qingdao), Qingdao, China; ^3^ National Demonstration Center for Experimental Fisheries Science Education, Shanghai Ocean University, Shanghai, China

**Keywords:** lncRNA, miRNA, mRNA, lncRNA-miRNA-mRNA ceRNA network, blind-side hypermelanosis, Chinese tongue sole

## Abstract

Blind-side hypermelanosis has emerged as a major concern in commercial rearing environments of the flatfish aquaculture industry. To date, the underlying molecular mechanisms are not well understood. To fill this gap, in this study, whole transcriptomic sequencing and analyses were performed using normal skins and hypermelanic skins of the blind side of Chinese tongue sole (*Cynoglossus semilaevis*). Differentially expressed long non-coding RNAs (DElncRNAs), miRNAs (DEmiRNAs), and differentially expressed genes as well as their competing endogenous RNA (ceRNA) networks were identified. A total of 34 DElncRNAs, 226 DEmiRNAs, and 610 DEGs were identified. Finally, lncRNA–miRNA–mRNA regulatory networks (involving 29 DElncRNAs, 106 DEmiRNAs, and 162 DEGs) associated with blind-side hypermelanosis were constructed. Gene Ontology (GO) and Kyoto Encyclopedia of Genes and Genomes (KEGG) enrichment analyses of 162 DEGs in ceRNA networks identified DEGs (e.g., oca2, mc1r, and ihhb) in pigmentation-related biological processes and DEGs (e.g., ca4, glul, and fut9) in nitrogen metabolism, glycosphingolipid biosynthesis, and folate biosynthesis pathways, as well as their corresponding DElncRNAs and DEmiRNAs to potentially play key regulatory roles in blind-side hypermelanosis. In conclusion, this is the first study on the ceRNA regulatory network associated with blind-side hypermelanosis in flatfish. These new findings expand the spectrum of non-coding regulatory mechanisms underpinning blind-side hypermelanosis, which facilitates the further exploration of molecular regulatory mechanisms of malpigmentation in flatfish.

## Introduction

Chinese tongue sole (*Cynoglossus semilaevis*) is a prevalent flatfish species in Chinese aquaculture because of its superior nutritive value and economic significance. Like other flatfishes, tongue sole will undergo morphological metamorphosis with an asymmetrical body plan during the larval stage to adapt to an over-substrate dwelling (benthic) lifestyle in later developmental stages (Chen et al., 2014; Shao et al., 2017; Fox et al., 2018; Lü et al., 2021). After metamorphosis, both eyes move to the same side (i.e., the ocular side), which becomes pigmented (black brown) while the blind side remains unpigmented (pure white) (Kang et al., 2011). However, under hatchery culturing conditions, hypermelanosis on the blind side after metamorphosis has increasingly become a concern in the production of flatfishes in Eastern Asian countries. Flatfishes with blind-side hypermelanic coat color are regarded as inferior products and are perceived as negative by consumers, which strongly limits their purchase desire. In certain cases, the proportion of hypermelanic individuals within aquaculture populations reaches as much as 90% (range 10–90%), resulting in a 10–15% drop of the market price (Li et al., 2021b).

Previous studies extensively focused on preventing or alleviating blind-side hypermelanosis by various aquaculture manipulations of flatfishes, such as roughing the floor of farming tanks with different substrates and modifying stocking density, lighting, and tank color (Iwata and Kikuchi, 1998; Amiya et al., 2005; Yamanome et al., 2005; Yamanome et al., 2007; Kang and Kim, 2012; Kang and Kim, 2013; Isojima et al., 2013; Nakata et al., 2017). Recently, studies in both tongue sole and Japanese flounder (*Paralichthys olivaceus*) that employed RNA sequencing and bioinformatics analysis found that hypermelanosis-related genes involved in tyrosine metabolism, melanogenesis, and thyroid synthesis pathways play important roles in blind-side hypermelanosis (Peng et al., 2020; Li et al., 2021a). Moreover, a recent study on Japanese flounder suggests that single-nucleotide polymorphism mutations are responsible for malpigmentation (Zhang et al., 2021). Current views support that the blind-side hypermelanosis trait in flatfish is controlled by polygenic genes, which can be affected by environmental factors; moreover, improvement by selective breeding is feasible. For example, a case study in tongue sole showed that hypermelanosis is a heritable variation (Li et al., 2021b). However, the genetic basis of a wider spectrum of gene regulatory networks behind hypermelanosis remains unclear.

Long non-coding RNAs (lncRNAs) are defined as a group of non-coding RNAs (ncRNAs) with transcripts longer than 200 nucleotides (Mattick and Rinn, 2015). They have been shown to play significant roles in various biological processes, such as cell development (Fatica and Bozzoni, 2014), transcriptional regulation (Bonasio and Shiekhattar, 2014), epigenetic modification (Mercer and Mattick, 2013), and immune response (Agliano, et al., 2019). LncRNAs can participate in translational and post-transcriptional regulation by acting as a microRNA (miRNA) sponge to counteract miRNA-mediated mRNA inhibition through competing endogenous RNA (ceRNA) networks (Denzler et al., 2016). Through these actions, lncRNAs can derepress the expression levels of target mRNAs by competitively binding to shared miRNA sequences (Kopp and Mendell, 2008; Salmena et al., 2011).

Recent studies showed that lncRNAs and miRNAs play important roles in skin color regulation. For example, Wan and Wang (2014) suggested that certain lncRNAs function in skin biology including epidermal development and keratinocyte differentiation. By employing genome-wide analysis, Ren et al. (2016) identified numerous lncRNAs associated with skin pigmentation in goats. Furthermore, evidence shows that lncRNAs can regulate melanogenesis via epigenetic regulation, while miRNAs can directly target many melanogenesis-related genes (Ho et al., 2020; review by Zhou et al., 2021). In aquaculture species, numerous significant differentially expressed lncRNAs (DElncRNAs) and miRNAs (DEmiRNAs) have been identified or further investigated by using different skin colors or other pigmented tissues in numerous species, such as Koi carp (*Cyprinus carpio* L.) (Luo et al., 2018, 2019; Dong et al., 2020; Yin et al., 2020), Pacific oyster (*Crassostrea gigas*) (Feng et al., 2018; Li Z. et al., 2021), red tilapia (*Oreochromis* sp. red tilapia) (Wang et al., 2018), common carp (*Cyprinus carpio*) (Yan et al., 2013), and Japanese flounder (*Paralichthys olivaceus*) (Wang et al., 2019). However, until today, the interactions among lncRNAs, miRNAs, and mRNAs in flatfish hypermelanosis remain unclear.

Accordingly, in the current study, high-throughput sequencing technology was used to identify and compare the lncRNA, miRNA, and mRNA expression profiles between blind-side hypermelanic and normal skin tissues in tongue sole. Based on conjoint multi-omics analyses, the lncRNA–miRNA–mRNA ceRNA regulatory network associated with blind-side hypermelanosis was characterized and verified for the first time in flatfish. The findings of this study provide new insight into the genetic basis underlying blind-side hypermelanosis and will facilitate the further investigation of molecular regulatory mechanisms of malpigmentation in flatfish.

## Materials and Methods

### Experimental Animals and Sample Collection

Tongue sole fish were obtained from Tangshan Weizhuo Aquaculture Co., Ltd., China. Briefly, nine blind-side hypermelanotic individuals (body weight 15 ± 5 g) with approximately 50% pigmented area were randomly selected and both hypermelanotic and normal skin tissue samples were collected. Then, two group samples were obtained, with three biological replicates, each replicate with three pooled pigmented/normal skin tissues from three different individuals. Sample names of blind-side hypermelanotic (BH) and normal (BN) skin were designated as BH-1, BH-2, BH-3, BN-1, BN-2, and BN-3, respectively. Skin tissues were immediately frozen and stored in liquid nitrogen until subsequent processing.

### RNA Isolation, Library Construction, and Sequencing

According to the instructions of the manufacturer’s manual, total RNA from skin tissues was extracted using Trizol reagent kit (Invitrogen, CA, USA). The purity of total RNA was assessed using a NanoPhotometer spectrophotometer, and the concentration was measured using the Qubit2.0 Fluorometer. The integrity was monitored by the Agilent 2100 bioanalyzer system (Agilent Technologies, Santa Clara, CA, USA). Ribosomal RNA (rRNA) was removed using the Ribo-zero rRNA Removal Kit (Epicentre, Madison, WI, USA). Then, the retained mRNAs and ncRNAs were fragmented into short fragments via fragmentation buffer and these fragments were reverse transcribed into first-strand complementary DNA (cDNA) with random primers. The second-strand cDNA was synthesized using DNA polymerase I, RNase H, dNTP (dUTP instead of dTTP), and buffer. Subsequently, cDNA fragments were purified with QiaQuick PCR extraction kit (Qiagen, Venlo, Netherlands), end repaired, poly(A) appended, and ligated to Illumina sequencing adapters. Finally, six strand-specific RNA libraries and six small RNA (sRNA) libraries were generated by amplifying the products via PCR; these were then sequenced based on the Illumina HiSeq 4000 platform by Gene Denovo Biotechnology Co. (Guangzhou, China).

### Differentially Expressed Gene Analysis

To obtain clean reads, fastp (v0.18.0) (Chen et al., 2018) and RNA-QC-Chain (Zhou et al., 2018) were used to remove low-quality bases and reads containing adaptors and polyA tails. Then, these clean reads were mapped to the tongue sole reference genome using HISAT2 (v2.1.0) (Kim et al., 2015). Finally, the mapped reads were merged using StringTie (v1.3.4) (Pertea et al., 2015). Gene expression levels were estimated based on fragments per kilobase of transcripts per million fragments mapped (FPKM) (Li and Dewey, 2011). Differentially expressed gene (DEG) analysis was performed by using DEseq2 software (Love et al., 2014). The genes with a false discovery rate (FDR) < 0.05 and |log_2_ (fold change)| > 1 were assigned as DEGs.

### Function Enrichment Analysis of DEGs

Gene Ontology (GO) and Kyoto Encyclopedia of Genes and Genomes (KEGG) pathway enrichment analyses were performed to identify the role of DEGs and DEG-related ncRNAs by using the GO-seq R package (Young et al., 2010) and KOBAS software (Mao et al., 2005), respectively. A *p*-value <0.05 was set as threshold to test significant differences.

### Identification of miRNAs

Clean tags with sRNAs were subject to GenBank database (release 209.0, https://www.ncbi.nlm.nih.gov/genbank/) and Rfam database (release 11.0, http://rfam.xfam.org/) to monitor and remove rRNA, scRNA, snoRNA, snRNA, and tRNA. Clean tags were then mapped to the reference genome of tongue sole to remove RNA fragments mapped to exons or introns by HISAT2 (v2.1.0) (Kim et al., 2015). All clean tags were then searched against the miRBase database (Release 22, http://www.mirbase.org/search.shtml) to identify known tongue sole miRNAs (existing miRNAs). Then, miRDeep2 software (Friedländer et al., 2012) was used to identify novel miRNAs. After tags were annotated as mentioned previously, the annotation results were determined following priority order: rRNA (etc.) > existing miRNA > existing miRNA edit > known miRNA > repeat > exon > novel miRNA > intron. The tags that could not be annotated as any of the aforementioned molecules were recorded as unannotated. According to the total miRNA expression in each sample, the miRNA expression level was calculated and normalized to transcripts per million. In addition, the expression of existing miRNAs, known miRNAs, and novel miRNAs was analyzed individually. Finally, DEmiRNAs were analyzed by edgeR software (Robinson et al., 2010) with adjusted *p*-value < 0.05 and |log_2_ (fold change)| > 1.

### Identification of lncRNAs

To identify lncRNAs, a strict stepwise pipeline was used. Raw sequencing data were first filtered by using fastp (v0.18.0) (Chen et al., 2018) with the following parameters: 1) removing reads containing adapters; 2) removing reads containing more than 10% unknown nucleotides; 3) removing low-quality reads containing more than 50% of low-quality bases (i.e., Q-value ≤ 20). Second, the obtained high-quality clean reads were mapped to rRNA database by the short reads alignment tool Bowtie2 (v2.2.8) to remove rRNA (Langmead and Salzberg, 2012). Third, paired-end clean reads of the remaining reads were mapped to the reference genome of tongue sole by HISAT2 (v2.1.0) (Kim et al., 2015) using “-rna-strandness RF” and other default parameters. Transcripts were reconstructed with the software StringTie (v1.3.4) (Pertea et al., 2015), and then these reconstructed transcripts were aligned to the reference genome and subjected to Cuffcompare software (Trapnell et al., 2010). With this procedure, novel transcripts and categories with transcripts longer than 200 bp and exon number above 2 were identified. Fourth, novel transcripts were subjected to Nr, KEGG, and GO databases to obtain protein functional annotation. The two software packages CNCI (Sun et al., 2013) and CPC (http://cpc.cbi.pku.edu.cn/) were used to predict the protein-coding potential of novel transcripts by default parameters. Intersected transcripts without coding potential were chosen as novel lncRNAs. The lncRNA types were identified according to their location relative to protein-coding genes. Finally, transcript abundances were quantified by the software StringTie (Pertea et al., 2015) using a reference-based approach. DElncRNA analyses between two groups were performed by DESeq2 (Love et al., 2014) with the parameter of FDR < 0.05 and |log_2_ (fold change)| > 1.

### Construction of ceRNA Network and Analysis

The three software packages mireap (http://sourceforge.net/projects/mireap/), miRanda (Pasquinelli, 2012), and TargetScan (http://www.targetscan.org/) were used to predict DEmiRNA targets. miRNA sequences and family information were obtained from the TargetScan website (http://www.targetscan.org/). According to miRNA prediction results, expression correlations between mRNA-miRNAs or lncRNA-miRNAs (Spearman rank correlation coefficient < −0.7) were identified as negative mRNA–miRNA pairs or lncRNA–miRNA pairs from DEGs, DEmiRNAs, and DElncRNAs. Expression correlation (Pearson correlation coefficient >0.9) between lncRNA-mRNA was selected as co-expressed lncRNA–mRNA pairs. Both mRNAs and lncRNAs in this pair were targeted and negatively co-expressed with a common miRNA. Hypergeometric cumulative distribution function test (*p*-value < 0.05) was performed to test whether common miRNA sponges between both genes were significant. Then, GO biological processes term and KEGG pathway analyses were performed to assess potential functions of mRNAs in the ceRNA network. Significantly enriched mRNAs (*p*-adjusted-value < 0.05 in GO analysis and *p*-value < 0.05 in KEGG analysis) and corresponding lncRNA–miRNA–mRNA pairs related to blind-side hypermelanosis were selected and the ceRNA network was constructed and visualized by Cytoscape software (http://www.cytoscape.org/).

### Validation of Transcriptome Sequencing

To verify the transcriptome sequencing result, three isoform genes (i.e., lncRNAs, miRNAs, and mRNAs) were randomly selected to perform real-time quantitative reverse transcription PCR (qRT-PCR) analysis. RNA samples originated from those used for library construction. Primer Premier 5 software was used for designing divergent primers. cDNA was synthesized by using the PrimeScript 1st strand cDNA Synthesis Kit (Takara, Tokyo, Japan). Then, qRT-PCR was performed with the 7500 Fast Real time PCR system (Applied Biosystems, USA) using the SYBR green I Premix Ex-Taq system (TaKaRa, Japan). actb2 actin was used as internal reference gene for mRNA and lncRNA qRT-PCR validation, and U6 was used as internal reference gene for miRNA qRT-PCR validation. The relative expression levels of DEmiRNAs, DElncRNAs, and DEGs were normalized by the 2^−ΔΔCT^ method (Livak and Schmittgen, 2001).

## Results

### Overview of Sequencing Data

To gain an overview of sRNAs of blind-side hypermelanotic and normal skin samples and to identify miRNAs that are potentially involved in blind side hypermelanosis mechanisms, six sRNA and six strand-specific libraries were separately constructed by using BH and BN samples, which were sequenced based on Illumina sequencing technology. Raw data have been deposited in the NCBI database under accession number PRJNA760350. The summary information of sRNA and strand-specific library sequencing data are shown in Supplementary Table S1 and Supplementary Table S2. After filtering, the clean reads/tags from each library were used to discern mRNAs, miRNAs, and lncRNAs.

**FIGURE 1:**
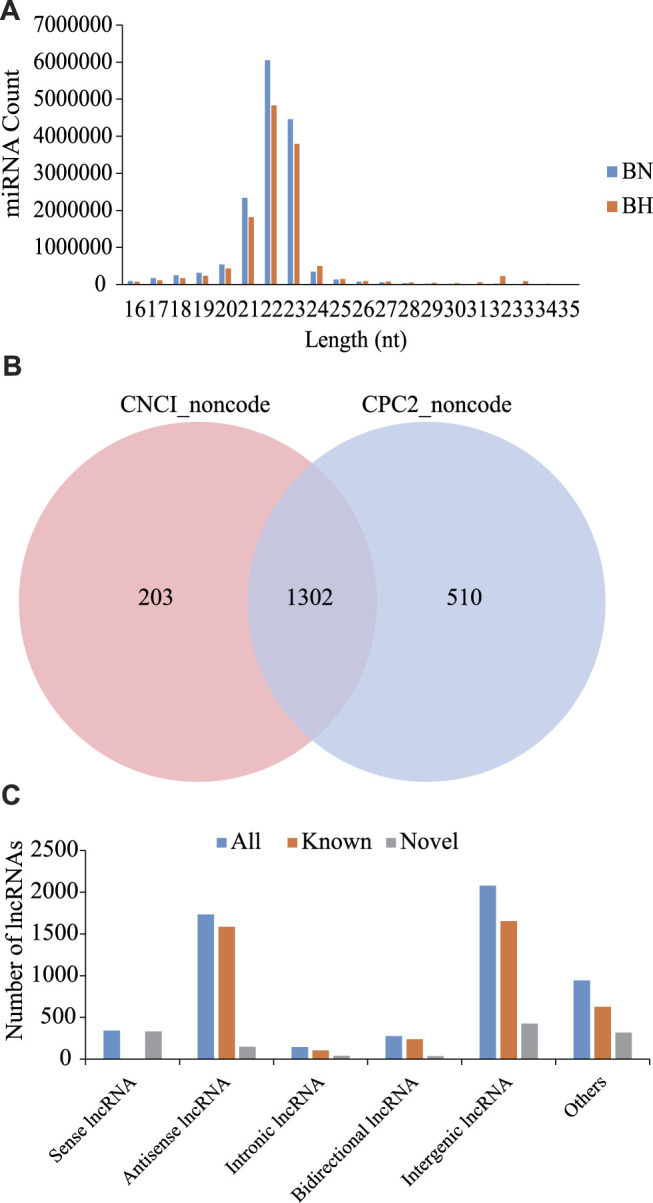
Signatures of miRNA and lncRNA sequencing data. **(A)** Size distribution of sRNAs from BN and BH libraries. **(B)** Venn diagrams of novel lncRNA prediction results based on CNCI and CPC2 software. **(C)** Distribution of lncRNA types.

As shown in Figure 1A, most sRNAs were distributed in length between 21 and 23 nt, with a peak value of 22 nt. After a series of screenings, a total of 971 miRNAs were identified (285 novel miRNAs and 686 known miRNAs). Based on CNCI and CPC software analyses and mapping results, 1,302 novel lncRNAs were predicted (Figure 1B) and 4,216 known lncRNAs were obtained. Furthermore, based on the position of these novel lncRNAs in the genome relative to protein-coding genes, they were divided into five categories, including 340 sense lncRNAs, 1734 antisense lncRNAs, 145 intronic lncRNAs, 276 bidirectional lncRNAs, and 2079 intergenic lncRNAs (Figure 1C).

### Identification of DEGs Associated With Hypermelanosis

**FIGURE 2:**
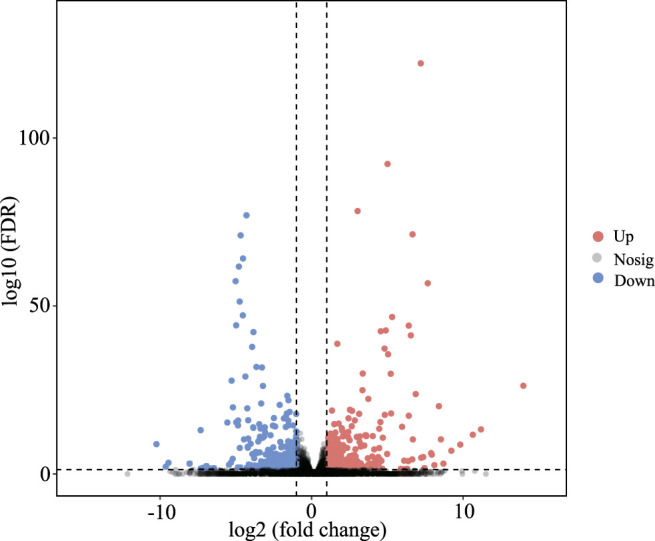
Volcano plots of differentially expressed genes (DEGs) between blind-side normal skin and blind-side hypermelanotic skin. FDR: false discovery rate.

In this study, by setting FDR < 0.05 and |log_2_ (fold change)| > 1 as cutoff values in the BN *versus* BH comparative groups, a total of 610 mRNAs were identified as DEGs, including 361 upregulated and 249 downregulated DEGs (Figure 2, Supplementary Table 3).

### GO and KEGG Enrichment Analyses of DEGs

**FIGURE 3:**
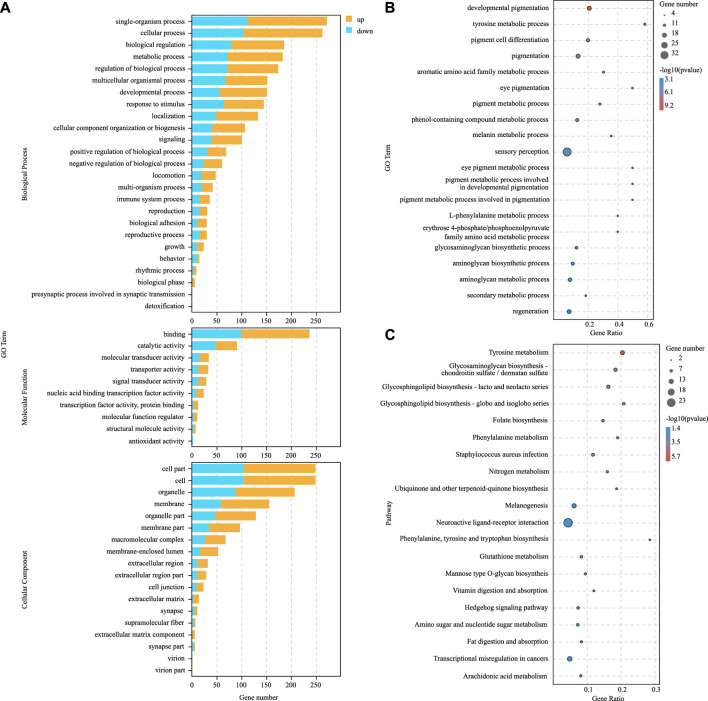
**(A)** Gene ontology (GO) enrichment analysis results for all DEGs between blind-side normal skin and blind-side hypermelanotic skin. **(B)** GO analysis results in the biological process for all DEGs between blind-side normal skin and blind-side hypermelanotic skin. **(C)** Kyoto Encyclopedia of Genes and Genomes (KEGG) pathway enrichment analysis results for all DEGs between blind-side normal skin and blind-side hypermelanotic skin.

To further explore the role of DEGs in blind-side hypermelanosis, enrichment analysis of GO and KEGG was conducted. For GO analysis, the dominant functions in each of the three main categories were single-organism process in the biological process categories, binding in the molecular function categories, and cell part in the cellular component categories (Figure 3A). Based on the GO term analysis, blind-side hypermelanosis may be associated strongest with the following biological processes (*p*-value < 0.05): sensory perception (GO:0007600), eye pigmentation (GO:0048069), pigmentation process (GO:0042440), neurological system process (GO:0050877), and developmental pigmentation (GO:0048066) (Figure 3B, Supplementary Table S4). In addition, DEGs were aligned against the KEGG pathways database to identify the pathways that were related to the blind-side hypermelanosis of *C. semilaevis*. The results showed that hypermelanosis-related DEGs were significantly enriched in 20 pathways (*p*-value < 0.05), including tyrosine metabolism, melanogenesis, glycosphingolipid biosynthesis, and folate biosynthesis (Figure 3C, Supplementary Table S5).

### Identification and Characterization of Hypermelanosis-Related miRNAs

**FIGURE 4:**
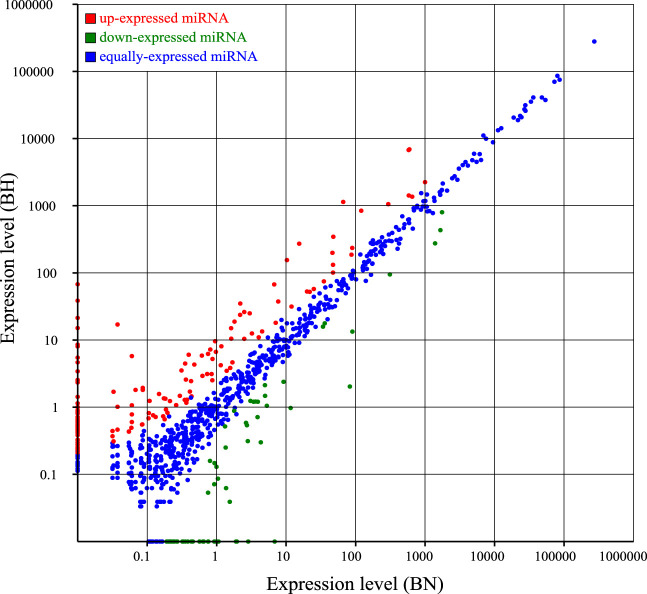
Scatter plots for differential expression miRNAs between blind-side normal skin (BN) and blind-side hypermelanotic skin (BH).

Compared with the blind-side normal skin group, 226 DEmiRNAs were identified in the blind-side hypermelanotic skin group, among which 155 were upregulated and 71 were downregulated (Figure 4, Supplementary Table S6). Furthermore, for constructing the miRNA–mRNA regulatory network, three software packages (mireap, miRanda, and TargetScan) were used to predict the DEmiRNAs target genes. Finally, a total of 2,274 mRNA–miRNA pairs were obtained, involving 215 miRNAs and 431 mRNAs (Supplementary Table S7). Certain miRNAs can combine with numerous different mRNAs, e.g., miR-2284 can bind to 41 mRNAs. Moreover, certain mRNAs were predicted to combine with multiple DEmiRNAs, e.g., NCBI_103382117 can link to 44 miRNAs.

### Identification and Characterization of Hypermelanosis-Related lncRNAs

**FIGURE 5:**
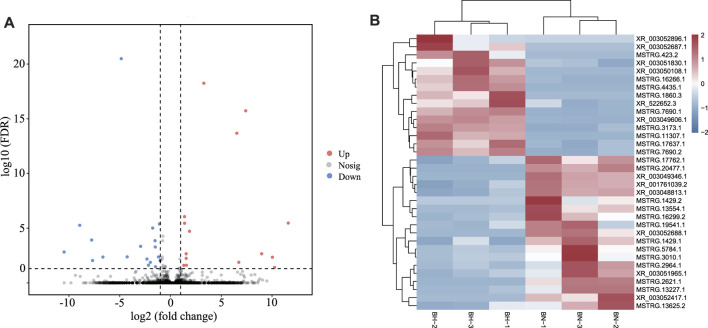
**(A)** Volcano plots for differentially expressed lncRNAs between BN and BH skin. **(B)** Heatmap of differentially expressed lncRNAs between BN and BH.

According to expression level comparison, a total of 34 DElncRNAs were identified (*p*-value < 0.05), including 15 up- and 19 downregulated DElncRNAs (Figure 5A, Supplementary Table S8). The heatmap (Figure 5B) demonstrates that both groups could be distinguished by their respective expression profiles. For lncRNA–miRNA network analysis, 274 lncRNA–miRNA pairs were predicted, involving 31 lncRNAs and 139 miRNAs (Supplementary Table S9).

### Construction and Visualization of ceRNA Network

**FIGURE 6:**
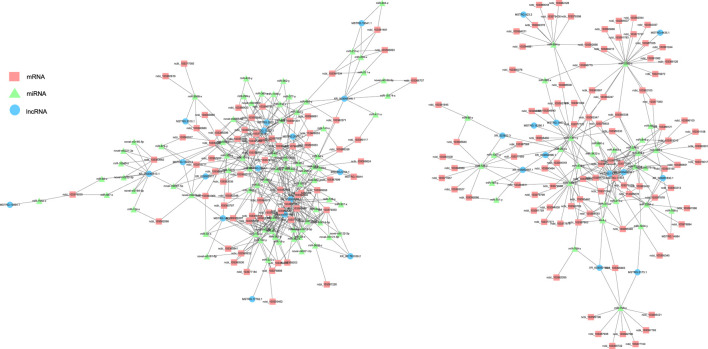
Functional lncRNA–miRNA–mRNA regulatory modules between blind-side normal skin and blind-side hypermelanotic skin. lncRNAs, miRNAs, and mRNAs are represented by blue circles, green triangles, and pink squares, respectively.

**FIGURE 7:**
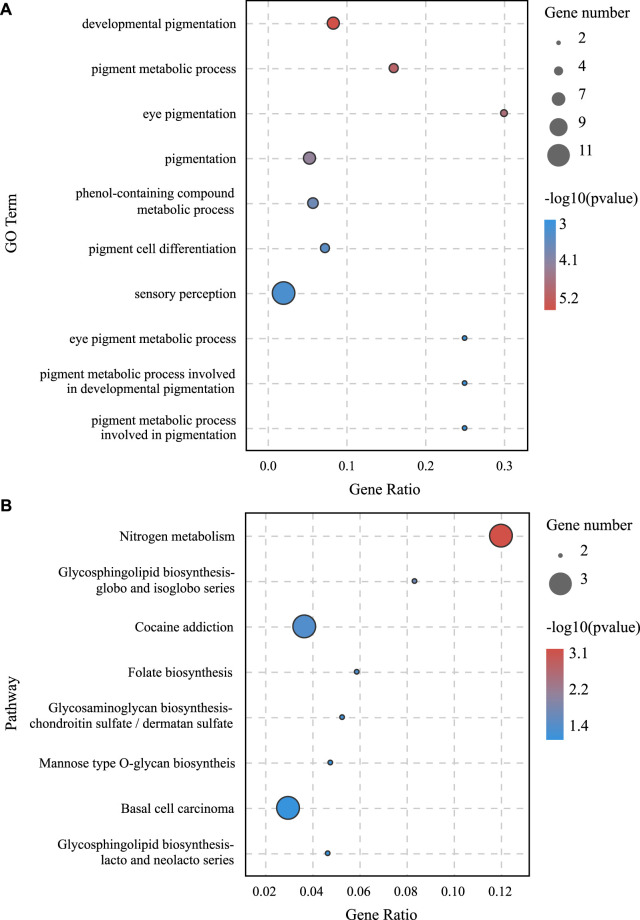
**(A)** GO enrichment analysis in the biological process for DEGs involved in the lncRNA–miRNA–mRNA network between BN and BH. **(B)** KEGG pathway enrichment analysis results for DEGs involved in lncRNA–miRNA–mRNA network between BN and BH.

lncRNA and mRNA share sequence homology, and miRNA can bind to mRNA following complementary base pairing. miRNA also likely binds with lncRNA that is homologous to its target gene sequence. Thus, a ceRNA hypothesis is proposed, where lncRNAs act as molecular sponges to adsorb miRNAs affecting target gene silencing caused by miRNAs. To explore the potential lncRNA–miRNA–mRNA regulatory network, the co-expression analysis of DElncRNAs and DEGs between BH and BN skins was conducted first. A total of 1,961 co-expression lncRNA–mRNA pairs were generated (Pearson correlation coefficient >0.9), including 31 lncRNAs and 430 mRNAs. Then, hypergeometric cumulative distribution function test was performed to select the final ceRNA pairs (*p*-value < 0.05), where 221 ceRNA pairs were obtained, involving 29 DElncRNAs, 106 DEmiRNAs, and 162 DEGs (Figure 6, Supplementary Table S10). Then, GO and KEGG enrichment analyses were performed to evaluate the 162 DEGs involved in the ceRNA regulatory network. The GO term enrichment analysis in biological process found a total of 144 significantly enriched GO terms (*p*-adjusted-value < 0.05) (Supplementary Table S11). The top 10 GO terms are presented in Figure 7A, including developmental pigmentation, pigment metabolic process, eye pigmentation, and pigment. GO analysis showed that certain DEGs, such as oca2, mc1r, ihhb, hps4, igsf11, and gpr143, might play important biological roles in blind-side hypermelanosis. Furthermore, KEGG analysis showed that there were eight enriched pathways with statistical significance (*p*-value < 0.05), such as nitrogen metabolism, glycosphingolipid biosynthesis, and folate biosynthesis, involving 16 DEGs, such as ca4, glul, fut9, pah, and gch1 (Figure 7B, Supplementary Table S12).

### qRT-PCR Validation of DElncRNAs, DEmiRNAs, and DEGs

**FIGURE 8:**
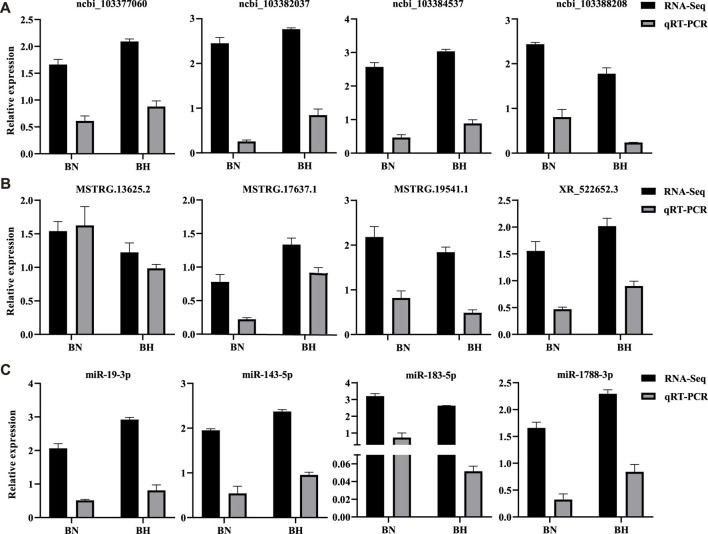
Real-time quantitative reverse transcription polymerase chain reaction (qRT-PCR) validation results of mRNAs, lncRNAs, and miRNAs. **(A)** Relative expression levels of four randomly selected DEGs from RNA-Seq and qRT-PCR. **(B)** Relative expression levels of four randomly selected differentially expressed lncRNAs from RNA-Seq and qRT-PCR. **(C)** Relative expression levels of four randomly selected differentially expressed miRNAs from RNA-Seq and qRT-PCR. Bars indicate standard errors.

Three isoform genes (lncRNAs, miRNAs, and mRNAs) were randomly selected to validate differential expression results of RNA-seq by qRT-PCR. The qRT-PCR results indicated identical trends with the RNA-seq results (Figure 8). All qRT-PCR primers are listed in Supplementary Table S13.

## Discussion

Improving the understanding of the regulatory network of blind-side hypermelanosis in tongue sole is valuable to uncover the underlying molecular mechanism and possible causes. To this end, in this study, the lncRNA, miRNA, and mRNA expression profiles of tongue sole blind-side hypermelanotic skins were systematically assessed and the results were compared with blind-side normal skins. To our knowledge, this is the first report on ceRNA regulatory networks associated with blind-side hypermelanosis in flatfish.

To better understand the hypermelanosis phenotype, the biological phenomenon of malpigmentation in flatfish should be described. Mainly two types of malpigmentation affect flatfish under hatchery rearing environments, i.e., ocular-side albinism and blind-side hypermelanosis (Venizelos and Benetti, 1999), both of which are of economic significance. According to previous studies, the mechanisms of albinism in flatfish can be ascribed to mutations in tyrosine metabolism-related genes (e.g., tyr and tyrp1), which have also been verified in other fishes and mammals (Arshad et al., 2018; Chi et al., 2006; Fistarol and Itin, 2010; Beirl et al., 2014; Klaassen et al., 2018; Pavan and Sturm, 2019; Jeong et al., 2020). However, the mechanisms underlying blind-side hypermelanosis in flatfish are not yet well understood. As mentioned previously, flatfish blind-side hypermelanosis may be a type of environmentally mediated regulation of gene expression and adaptation, where the ion types and/or concentrations in seawater may play important roles (Li et al., 2021a). Indeed, in fish, numerous studies have shown that external environmental short- and long-term stimuli may result in physiological and morphological skin color changes, respectively (Fujii, 2000; Sugimoto et al., 2005; Aspengren et al., 2009; Kelsh et al., 2009). These are mediated by pigment organelle aggregation or dispersion within skin chromatophores, as well as apoptosis or proliferation of skin chromatophores, respectively (Cal et al., 2017). Furthermore, it is worth emphasizing that (ctenoid) scales are absent in ocular-side albinistic parts (no scale), while in blind-side hypermelanotic parts, ctenoid scales cannot transform into cycloid scales at the adult stage. Consequently, pigment cells accumulate in attached skins. This explained the generation of blind-side hypermelanosis.

The essence of this phenomenon can be explained based on predicted lncRNA–miRNA and miRNA–mRNA pairs. A global lncRNA–miRNA–mRNA network was constructed, involving 29 DElncRNAs, 106 DEmiRNAs, and 162 DEGs. The results of GO and KEGG analysis showed that multiple biological processes associated with pigmentation were remarkably enriched. To some extent, these findings are consistent with previous studies in other abnormally pigmented fishes (Zou et al., 2015; Zhu et al., 2016; Zhang et al., 2017; Gan et al., 2021). Clearly, the pigmentation of skin is biologically associated with processes related to pigment metabolism. However, in the current study, processes related to neurological system, ion transport, ectodermal placode formation, and sensory perception were also significantly enriched.

The integration of phenotypic characteristics and regulatory network analysis to identify signatures of blind-side hypermelanosis clarified the newly identified key biological processes that seem to be important for blind-side pigmentation. Therefore, it seems reasonable to speculate that non-coding elements (e.g., lncRNAs and miRNAs) directly regulate genes enriched in processes related to neurological system, ion transport, ectodermal placode formation, and sensory perception. Likely, unfavorable environmental stresses alter the expression profiles of key genes, which are mediated by lncRNAs and miRNAs. This is consistent with previous reports on other aquaculture species, where numerous lncRNAs and miRNAs have been confirmed to play key roles in pigmentation regulation (Feng et al., 2018; Luo et al., 2018; Luo et al., 2019; Dong et al., 2020; Yin et al., 2020; Li Z. et al., 2021).

In combination with the results of previous studies, the results obtained in this study indicate a complex regulatory network response to hypermelanosis, suggesting that fish–environment crosstalk regulates the response or adaption to stress. The transcriptomic signatures of blind-side pigmentation are diverse in nature, which is in accordance with the polygenic architecture associated with moderate heritability (Li et al., 2021b). In China, tongue sole was domesticated and cultured by mass spawning in 2002 using a wild-caught population. Since then, blind-side hypermelanosis has evolved from sporadic cases with limited ratio of pigmented areas and ratio of melanistic individuals, to more common cases as described previously (Li et al., 2021b). This is likely because the artificial habitats are different from the wild. The habitat changes including the substrate in commercial rearing tanks (which is smoother), diets, light levels, and stocking density. Extensive clues can be found in case studies on rodents’ coloration (see review by Hoekstra, 2006). Therefore, it seems that blind-side hypermelanosis in flatfish may imply an adaptive morphology change. In tongue sole, there is no morphological transformation in the scales of pigmented parts (from ctenoid scales to cycloid scales) during the grow-out period. Being richly endowed with ctenoid scales in blind-side pigmented sections of the body can help fish to cope with the relative smooth substrate in tanks. As flatfishes have a bottom-dwelling lifestyle, rough substrates like sands make flatfishes to quickly bury themselves into sands in the wild. Similar implications have been reported for the Japanese flounder, where sandy substrates can alleviate blind-side hypermelanosis (Iwata and Kikuchi, 1998). It cannot be ruled out that blind-side malpigmentation in tongue sole is associated with genomic signatures of artificial selection (for growth and disease resistance traits).

**FIGURE 9:**
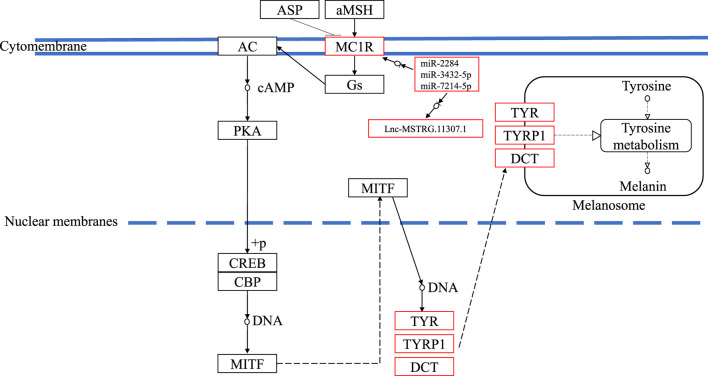
The underlying ceRNA mechanism of blind-side hypermelanosis in melanogenesis pathway in tongue sole.

In this study, compared with blind-side normal skins, non-coding transcripts in blind-side pigmented skins changed substantially. These interesting findings support the perspective that mutations in regulatory regions can be regarded as a mechanism of phenotype fine tuning (Hoekstra, 2006). For instance, the mc1r gene is extensively involved in several key biological processes mentioned previously and in the melanogenesis pathway. Mc1r was significantly upregulated in tongue sole blind-side pigmented skin (by 3.56-fold). However, the expression levels of mc1r in other species were not significantly different between white and pigmented skin samples (Peñagaricano et al., 2012; Bradley et al., 2013; Zhang et al., 2015; Chen et al., 2020). Thus, the corresponding lncRNA (i.e., MSTRG.11307.1) and miRNAs (i.e., miR-2284, miR-3432, and miR-7214) of mc1r are worthy of further investigation. The underlying mechanism of how ceRNA functioned in the melanogenesis pathway is outlined in Figure 9. Despite the clear value of these findings, more research is needed.

## Conclusion

In this study, 34 DElncRNAs, 226 DEmRNAs, and 610 DEGs associated with blind-side hypermelanosis were identified in tongue sole. The ceRNA (lncRNA–miRNA–mRNA) regulatory network analysis highlights that lncRNAs and miRNAs play critical regulatory roles in blind-side hypermelanosis. Follow-up studies specifically targeted at investigating the functional roles of these regulatory elements in the response to blind-side hypermelanosis could help to further uncover the underlying molecular mechanism. This may further contribute to improving this trait by selective breeding in flatfish.

## Data Availability

The datasets presented in this study can be found in online repositories. The names of the repository/repositories and accession number(s) can be found in the article/Supplementary Material.
